# Calcium ion regulation of sodium alginate in pure buckwheat noodles shown by *in vitro* simulated digestion

**DOI:** 10.3389/fnut.2022.1105878

**Published:** 2023-01-12

**Authors:** Hongyan Wang, Jiukai Zhang, Lingyu Han, Jijuan Cao, Jixin Yang, Ying Zhang, Bing Hu

**Affiliations:** ^1^Key Laboratory of Biotechnology and Bioresources Utilization, College of Life Sciences, Ministry of Education, Dalian Minzu University, Dalian, China; ^2^Chinese Academy of Inspection and Quarantine, Beijing, China; ^3^Collaborative Innovation Center of Provincial and Ministerial Co-construction for Marine Food Deep Processing, Dalian Polytechnic University, Dalian, China; ^4^Faculty of Arts, Science, and Technology, Wrexham Glyndwr University, Wrexham, United Kingdom

**Keywords:** buckwheat noodles, sodium alginate, calcium ions, *in vitro* simulated digestion, release of glucose

## Abstract

The effects of calcium sodium alginate on quality and starch digestion of pure buckwheat noodles were investigated. The incorporation of calcium ions into noodles containing sodium alginate was found to reduce water absorption by the noodles during cooking, together with an increase of the turbidity. Calcium addition improved the noodle texture, as shown by the measurement of hardness, elasticity, adhesion, and chewability. *In vitro* simulations of digestion showed that calcium ion cross-linking with sodium alginate reduced glucose formation by approximately 23.3 mg/g. X-ray diffraction and Fourier transform infrared spectroscopy showed alterations in the crystal zone of the noodles induced by an alginate gel network, although no new chemical substances were generated. Noodles prepared by this exogenous method may be useful as functional foods for patients with diabetes.

##  1. Introduction

Starch is an essential carbohydrate component of the human diet. Hydrolysis by amylase reduces the starch to glucose that can then be utilized by the body. However, starch, especially in the form of processed foods, tends to be rapidly digested, leading to potential health issues such as obesity and diabetes, and thus people are becoming more aware of the importance of consuming healthy food (1, 2).

Noodles are a staple food in many Asian countries and have become increasingly popular among consumers around the world (3). However, noodles have a high glycemic index (GI) indicating that they are easily hydrolyzed (4). It is thus important to attempt to reduce the GI of noodles. Buckwheat and its processed products contain a variety of bioactive substances, including specific polysaccharides, dietary fiber, and polyphenols, all of which are of great nutritional values (5). Therefore, buckwheat noodles have attracted increased consumer attention (6). It has been found that buckwheat noodles can slow down sugar release resulting from starch digestion *in vitro* and that the addition of 45% Tartary buckwheat to noodles can be beneficial to human health (7).

It is well-documented that adding functional components to starchy foods can slow the rate of digestion (8–10). Sodium alginate is a natural polysaccharide obtained from brown algae. It is a linear compound composed of (1→4)-linked β-D-mannuronic acid and α-L-guluronic acid pyranose residues (11). Sodium alginate, being highly hydrophilic, has good hygroscopic properties and dissolves readily in both hot and cold water where it rapidly forms a viscous colloidal solution with strong activity. It can also incorporate divalent metal ions (excluding mercury and magnesium) to form an alginate gel of high tensile strength within the food (12–14). Muhammad Lubowa et al. prepared rice noodles with pre-gelatinized high amylose corn starch combined with Ca^2+^ induced sodium alginate, which solidified alginate, improved the tensile strength of noodles, and made the noodles more dense (15). Similarly, Masahiro Yuasa et al. used a plastic syringe to add mentsuyu containing 1% (w/w) sodium alginate to 5% (w/w) calcium lactate solution in a drop by drop manner. The solution immediately gelled to form mentsuyu caviar, which gave mentsuyu and caviar an even more striking visual appearance (16).

However, the effect of Ca^2+^ induced sodium alginate gel on the quality and digestive characteristics of buckwheat noodles has not been systematically studied. In this study, two methods of Ca^2+^ incorporation into sodium alginate and its effects on the digestion of pure buckwheat noodles were investigated. The first method was exogenous, where Ca^2+^ permeated sodium alginate-containing noodles from the outside during cooking, resulting in the formation of a stable gel network. The second method was endogenous, where the Ca^2+^ was released from calcium carbonate after acid treatment and subsequent cross-linking with sodium alginate to form the gel network during the procedure of making the noodles. Here, the effects of these different methods of Ca^2+^ incorporation into the sodium alginate networks on the digestion of the noodles were studied, measuring parameters associated with cooking, texture, and *in vitro* digestion of the noodles. In addition, the effects of calcium and sodium alginate cross-linking on the crystal structure of the starch were assessed by X-ray diffraction (XRD) and Fourier transform infrared spectroscopy (FTIR). The results indicate the preferable methods for the manufacture of functional noodles.

## 2. Materials and methods

### 2.1. Materials

Pure buckwheat flour was provided by Dalian Hongrun Whole Grain Food Co., Ltd., (Dalian, China). Pepsin (≥ 3,800 U/g), trypsin (1:4000), and amyloglucosidase (15 U/mL) were purchased from Shanghai Yuanye Bio Biotechnology Co., Ltd., (Shanghai, China). Other reagents were obtained from Beijing Chemical Reagent Co., (Beijing, China). All reagents were of analytical grade.

### 2.2. Methods

#### 2.2.1. Preparation of noodles

Preparation of pure noodles: the noodles were prepared by mixing 90 g of pure buckwheat flour with 50 g of deionized water. The optimal cooking time was 3 min 40 s. It’s as the control group.

Exogenous method: 90 g of pure buckwheat flour was mixed with 50 g of sodium alginate solution (0.1–0.5%) using a mixer (HMJ-A35M1, Guangdong, China) for 15 min. After allowing the dough to rest for 15 min, it was sheeted using a noodle machine (MR-08, Guangdong, China) and the sheets were cut into noodles 6.0 mm wide and 2.0 mm thick. Then use the prepared CaCl_2_ solution (0.3–0.5 M) to cook the noodles until the optimal cooking time: 3 min and 40 s.

Endogenous method: 90 g of pure buckwheat flour was mixed with CaCO_3_ powder (3–9% of the dough) and added to 50 g sodium alginate solution (0.1–0.5%) using a mixer for 15 min. After allowing the dough to rest for 15 min, it was sheeted using a noodle machine and the sheets were cut into noodles 6.0 mm wide and 2.0 mm thick. The noodles were cooked with the citric acid solution, pH 4.0, until the optimal cooking time: 3 min and 40 s.

#### 2.2.2. Cooking characteristics of noodles

In a separate experiment, the characteristics of the noodles were evaluated according to the method of Gimenez et al. (17) with slight modifications.

Water absorption of noodles: Ten pieces of noodles were weighed on a balance and the weight was recorded as M_1_. The noodles were then boiled in 500 ml of deionized water at 160°C until cooked. The noodles were then immediately removed from the water, washed with cool water for 10 s, and placed on a screen mesh. After standing at room temperature for 5 min, the noodles were weighed, with the weight recorded as M_2_. The rate of water absorption was calculated using the equation below and the experiment was repeated three times.


(1) $$\mathrm{Water}\mathrm{absorption}\left(\%\right)=\left(\frac{M_{2}-M_{1}}{M_{1}}\right)\times 100\%$$

Turbidity: the cooking water was then cooled and transferred to a 500 ml volume bottle, which was reached volume with deionized water, shaken and left for 2 h, and the absorbance at 460 nm measured by ultraviolet spectrophotometer is turbidity. Measurements were obtained in triplicate.

#### 2.2.3. Texture profile analysis

The texture profile analysis (TPA) of the noodles was conducted with a texture analyzer (TA-XTC, Boson Tech Co., Ltd., China) (18). The parameters used for the analysis were as follows: P/5 probe, pre-test speed = 2.0 mm/s; test speed = 0.8 mm/s; post-test speed = 0.8 mm/s; compression degree = 50%. Samples were analyzed in octuplicate.

#### 2.2.4. Starch digestion *in vitro*

The digestibility of the noodles was analyzed *in vitro* as described by Englyst et al. with minor modifications (19). The cooked fresh noodles were ground and the sample (3 g) was mixed with 30 mL of distilled water in a beaker and stirred using a magnetic stirrer at 37°C for 10 min. Pepsin (1.0 mL) was then added and stirred for 30 min to simulate gastric digestion (pH = 2). Once completed, an aliquot (1.0 mL) was withdrawn (time 0) and added to 4 mL absolute alcohol to stop any further enzyme reaction. Amyloglucosidase (0.1 mL) was then added to the beaker to prevent inhibition of the end products of pancreatic α-amylase. Then 1 ml 5% trypsin was added to represent ileal digestion. Aliquots (1.0 mL) were removed at different times (20, 30, 60, 90, 120, and 180 min), which were inactivated by the addition of absolute ethanol (4.0 mL). Subsequently, the glucose content was determined by the 3,5-dinitrosalicylic acid (DNS) method (20). Absorbances at 540 nm were measured using a UV-3600 spectrophotometer (Shimadzu, Kyoto, Japan) and the amount of hydrolyzed sugar was calculated as follows:

C⁢H⁢O=C×D×(V-S)

where,

CHO: amount of hydrolyzed sugar generated in the whole system during digestion *in vitro* (mg);
C: standard amount of glucose detected from the standard operating curve (mg);
D: dilution ratio of dialysis solution;
V: total volume of the solution for digestion of the whole system *in vitro* (mL);
S: volume of solution taken from the system each time (mL).

#### 2.2.5. X-ray diffraction analysis

The cooked noodles were immediately frozen in a −80°C freezer. The noodle samples were lyophilized, ground to powder (SCIENTZ 18N, Zhejiang Side Equipment Co., Ltd., Zhejiang, China) and passed through a 60-mesh sieve before X-ray diffraction analysis. X-ray diffraction analysis of the noodles was conducted using an XRD-6000 diffractometer (Shimadzu) with an operating voltage of 40 kV and a current of 30 mA. The diffraction scan angle (2θ) ranged from 5 to 50° with a scanning speed of 2°/min.

#### 2.2.6. FTIR analysis

The sample preparation was in a similar manner to the X-ray diffraction analysis. Samples were ground to a fine powder and were mixed with dry potassium bromide (1:100, w/w) and tableted at 10 000 PSI. Spectra were recorded in an IR-Affinity-1 spectrophotometer (Shimadzu) between 399 and 4,000 cm^–1^ at a resolution of 2 cm^–1^.

#### 2.2.7. Statistical analysis

All experiments were performed with three replicates. Data were expressed as mean ± standard deviation and analyzed using Origin 2021 (Origin-Lab, Inc., USA) and GraphPad Prism 8.4.0 (GraphPad Software, LLC, USA) for Windows. Differences between means were assessed by one-way analysis of variance (ANOVA) followed by Duncan’s test.

## 3. Results and discussion

### 3.1. Cooking characteristics of noodles

The results of the water absorption and turbidity of noodles prepared by the exogenous method are shown in Table 1. Water absorption is defined as the ability of the noodles to retain water, and is controlled mainly by the strength of the network formed by starch, fiber, or protein (21). Compared with the control groups in both exogenous and endogenous methods, the water absorption of noodles supplemented with sodium alginate colloid was significantly reduced, and further decreases were seen in the noodles in which calcium ions had been incorporated with the sodium alginate (*p* < 0.05). This may be because the capacity for water absorption is related to the integrity of the structural network, and a highly cross-linked network structure limits water absorption (22). The noodles cooked by the exogenous method are more turbid than the noodle soup of the control group. The possible reason is that during the cooking process, part of calcium chloride entered the noodles and combined with sodium alginate to form a network structure, while the other part of calcium chloride remained in the noodle soup, increasing the turbidity degree of the noodle soup. However, different from the exogenous method, the turbidity of noodles prepared by the endogenous method decreased with the increase of the concentrations of sodium alginate and calcium carbonate, which may be caused by the increase of the concentration of both, which further promoted the formation of gel network structure.

**TABLE 1:** Cooking characteristics of noodle samples.

Method	Noodle samples	Water absorption (%)	Turbidity
Exogenous method	Control (H_2_O)*	131.727 ± 3.285^a^	0.069 ± 0.002^g^
	0.1% Sodium alginate (H_2_O)*	119.330 ± 5.064^b^	0.092 ± 0.031^fg^
	0.1% Sodium alginate (0.3M CaCl_2_)*	109.287 ± 5.500^de^	0.156 ± 0.002^bc^
	0.1% Sodium alginate (0.4M CaCl_2_)*	109.537 ± 2.577^cde^	0.208 ± 0.001^a^
	0.1% Sodium alginate (0.5M CaCl_2_)*	102.009 ± 0.299^e^	0.160 ± 0.002^bc^
	0.3% Sodium alginate (H_2_O)*	116.808 ± 14.879^bc^	0.109 ± 0.001^ef^
	0.3% Sodium alginate (0.3M CaCl_2_)*	112.768 ± 1.935^bcd^	0.148 ± 0.003^bcd^
	0.3% Sodium alginate (0.4M CaCl_2_)*	111.246 ± 0.857^bcde^	0.131 ± 0.007^cde^
	0.3% Sodium alginate (0.5M CaCl_2_)*	106.494 ± 1.783^de^	0.163 ± 0.007^bc^
	0.5% Sodium alginate (H_2_O)*	117.858 ± 1.224^bc^	0.101 ± 0.002^g^
	0.5% Sodium alginate (0.3M CaCl_2_)*	116.906 ± 4.045^bc^	0.118 ± 0.014^def^
	0.5% Sodium alginate (0.4M CaCl_2_)*	108.145 ± 2.916^cde^	0.112 ± 0.001^ef^
	0.5% Sodium alginate (0.5M CaCl_2_)*	105.210 ± 2.806^de^	0.174 ± 0.004^b^
Endogenous method	Control (Citric acid)*	133.759 ± 0.858^a^	0.074 ± 0.003^g^
	0.1% Sodium alginate (Citric acid)*	133.501 ± 3.289^a^	0.147 ± 0.004^b^
	0.1% Sodium alginate/3% CaCO_3_ (Citric acid)*	129.970 ± 5.575^ab^	0.143 ± 0.005^b^
	0.1% Sodium alginate/6% CaCO_3_ (Citric acid)*	123.784 ± 1.967^bcd^	0.140 ± 0.003^b^
	0.1% Sodium alginate/9% CaCO_3_ (Citric acid)*	117.172 ± 1.497^de^	0.119 ± 0.007^cd^
	0.3% Sodium alginate (Citric acid)*	123.001 ± 0.055^bcde^	0.123 ± 0.006^c^
	0.3% Sodium alginate/3% CaCO_3_ (Citric acid)*	119.832 ± 9.728^cde^	0.118 ± 0.003^cd^
	0.3% Sodium alginate/6% CaCO_3_ (Citric acid)*	117.986 ± 8.109^de^	0.118 ± 0.004^cd^
	0.3% Sodium alginate/9%CaCO_3_ (Citric acid)*	115.233 ± 2.219^e^	0.115 ± 0.002^cd^
	0.5% Sodium alginate (Citric acid)*	132.062 ± 1.120^a^	0.156 ± 0.004^a^
	0.5% Sodium alginate/3%CaCO_3_ (Citric acid)*	127.251 ± 2.171^abc^	0.113 ± 0.005^d^
	0.5% Sodium alginate/6% CaCO_3_ (Citric acid)*	121.569 ± 1.825^cde^	0.105 ± 0.002^e^
	0.5% Sodium alginate/9% CaCO_3_ (Citric acid)*	114.959 ± 3.357^e^	0.087 ± 0.003^f^

*Represents different solution environments for cooking noodles.

Results are presented as means ± standard deviations. Lowercase letters within columns represent significant differences (*P* < 0.05) between samples.

### 3.2. Textural properties of noodles

**FIGURE 1:**
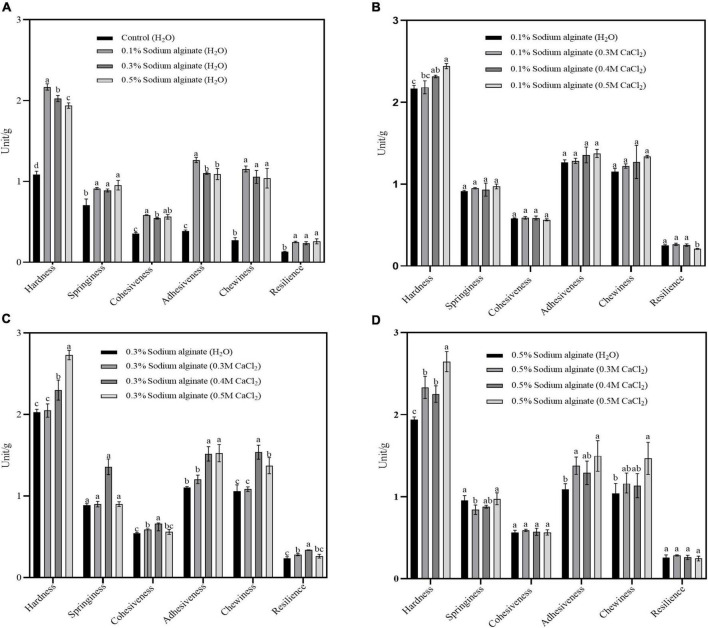
**(A–D)** Show the effects of varying Ca^2+^ concentrations on the textural properties of buckwheat noodles prepared by the exogenous method. Control group panel **(A)** was steamed with deionized water. The experimental group in panels **(B–D)** were steamed with calcium chloride solution of different concentrations.

**FIGURE 2:**
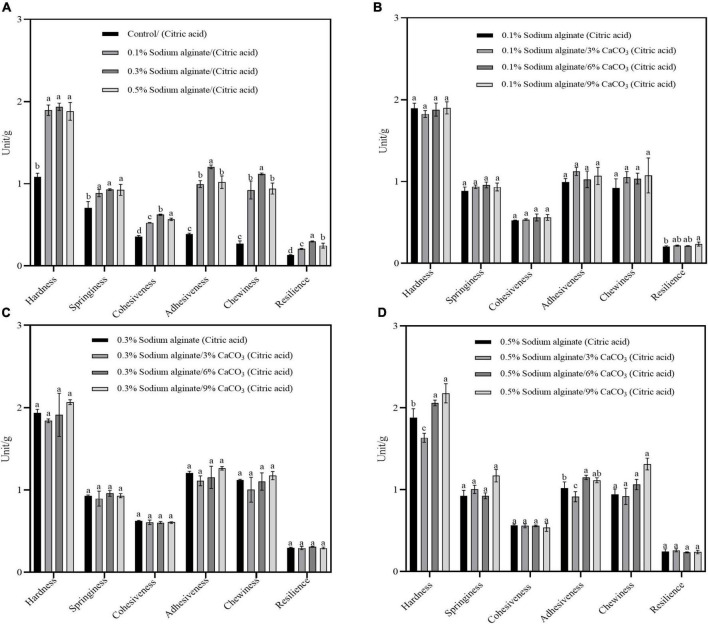
**(A–D)** Show the effects of Ca^2+^ concentrations of the textural properties of noodles prepared by the endogenous method. The solution environment for cooking noodles in panels **(A–D)** is 0.1 g/L citric acid solution (pH = 4).

Figure 1 illustrates the textural properties of the noodles. It was found that, compared with the control noodles, the hardness, springiness, cohesiveness, adhesiveness, chewiness, and other textural properties were enhanced by the addition of sodium alginate at different concentrations (Figure 1A). The hardness, adhesiveness, and chewiness of noodles with Ca^2+^ incorporated into the sodium alginate by the exogenous method were significantly higher than those in the sodium alginate only groups (Figures 1B–D). The increase in these properties may be due to the effective cross-linking of Ca^2+^ with sodium alginate, resulting in the strengthening of the three-dimensional structures. However, compared with the exogenous method, there were no significant differences in the textural characteristics between the noodles prepared by the endogenous method with Ca^2+^ and the sodium alginate only groups (Figures 2A–D), which may be due to the fact that citric acid is a weak electrolyte with weak ionizing ability, and the reaction with calcium carbonate releases less calcium ions, resulting in a poor ability to bind sodium alginate and not form a more stable strong gel. These results confirm that the gel network structure of Ca^2+^ and sodium alginate formed by the exogenous method could improve the texture of noodles.

### 3.3. Digestibility of noodles *in vitro*

**FIGURE 3:**
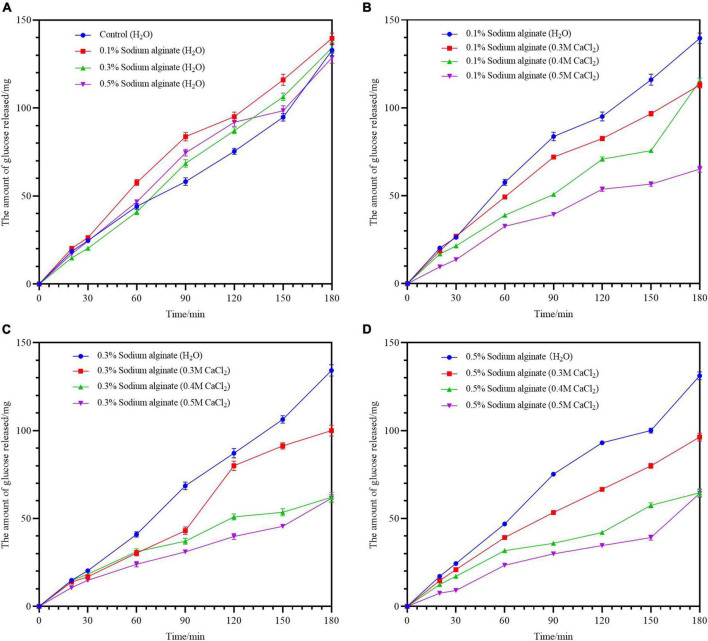
**(A–D)** Show the effects of varying Ca^2+^ concentrations of the digestibility of buckwheat noodles prepared by the exogenous method. Control group panel **(A)** was steamed with deionized water. The experimental groups panels **(B–D)** were steamed with calcium chloride solution of different concentrations.

Figure 2 shows the amounts of reduced sugars released over 180 min of *in vitro* digestion for all the noodle samples. The same trend of starch hydrolysis was seen in all samples, with the amount of glucose released increasing gradually over time. Compared with the control noodles, the addition of different concentrations of sodium alginate (0.1–0.5%) did not reduce the glucose release (Figure 3A). However, the incorporation of Ca^2+^ into the sodium alginate-containing noodles using the exogenous method resulted in reductions in glucose release, with noodles containing higher concentrations of Ca^2+^ showing lower glucose release; the maximum reduction in glucose release was approximately 23.3 mg/g (Figures 3B–D). This is likely the consequence of the denser network formed by the calcium and sodium alginate, preventing the leaching of internal molecules. These findings indicate that the network composed of sodium alginate and calcium ions can protect the starch from amylase hydrolysis. The concomitant use of higher concentrations of Ca^2+^ results in a denser gel network that provides a better barrier to enzymatic hydrolysis. These results are consistent with previous findings where Ca^2+^ was used for cross-linking with sodium alginate to form a core-shell structured macrocapsule calcium alginate (23).

**FIGURE 4:**
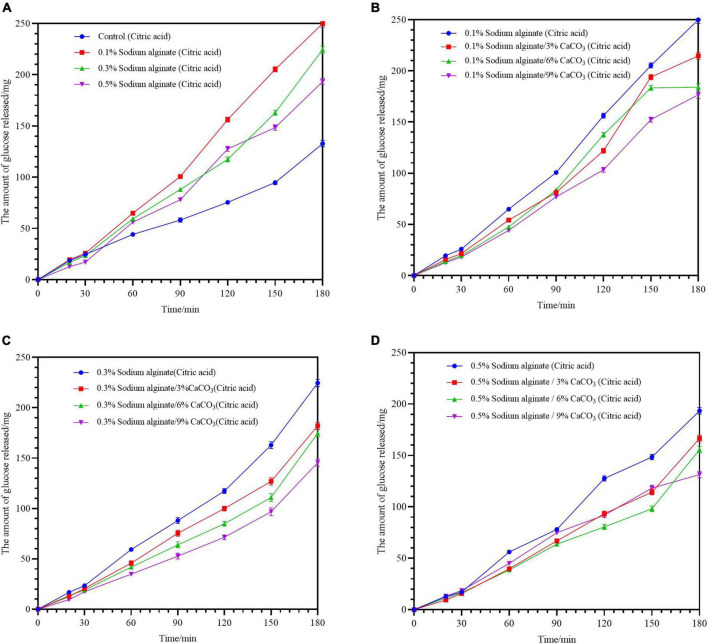
**(A–D)** Show the effects of varying Ca^2+^ concentrations on the digestibility of buckwheat noodles prepared by the endogenous method. The solution environment for cooking noodles in panels **(A–D)** is 0.2 g/L citric acid solution (pH = 4).

The glucose release of noodles prepared by the endogenous method of calcium ion combined with sodium alginate is shown in Figures 4A–D. The glucose release was lower than that in the sodium alginate group but was not decreased in comparison with the control group. Although increased Ca^2+^ concentrations were beneficial to glucose release compared with the sodium alginate only groups, the incorporation of calcium did not significantly reduce the glucose release compared with the control group. The likely explanation for this phenomenon may be as below. On the one hand, the relatively low concentrations of calcium ions were released by the reaction of calcium carbonate with acid, resulting in the formation of a weaker gel structure. On the other hand, the carbon dioxide formed during the reaction may affect the structure of the dough, creating greater looseness, which may also account for the poorer textural properties.

### 3.4. XRD patterns of starches

**FIGURE 5:**
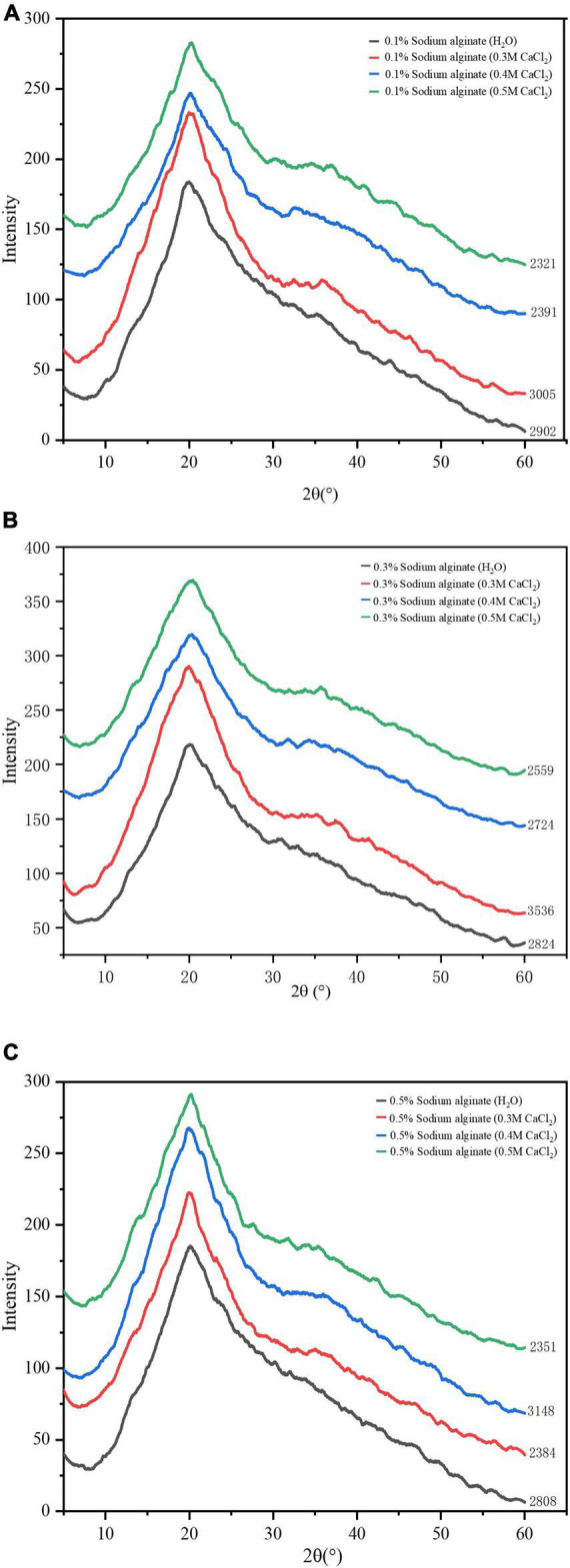
**(A–C)** Show the X-ray diffraction patterns of sodium alginate with the addition of varying Ca^2+^ concentrations by the exogenous method.

X-ray diffraction (XRD) was used to investigate changes in the crystallinity of noodles prepared by the exogenous method. The XRD patterns of all the samples are summarized in Figure 5. The crystallinity of foods is a significant determinator of their physical properties, and affects digestibility (24). Starch is composed of four types of crystal structures, namely, A, B, C, and V (25). As seen in the XRD patterns in the figure, clear peaks are visible at 2θ of 20°, which indicate the possible presence of an amylose-lipid complex (V-type) in the starch particles and endows the starch with properties such as resistance to digestion and improved food texture (26). The XRD patterns of the noodles with sodium alginate and calcium were similar to those of the sodium alginate only noodles; however, the area of the peak was altered by the addition of calcium ions. The X-ray diffraction pattern results of the endogenous method ara similar to those of the exogenous method, which only changed the crystal area of the noodles, which are not mentioned here. This indicates that the crystal structure of the starch was not changed by the cross-linking of calcium ions with sodium alginate, with the addition of calcium altering only the crystal area of the noodles, possibly affecting the quality of the noodles (27).

### 3.5. FTIR spectroscopy of starches

**FIGURE 6:**
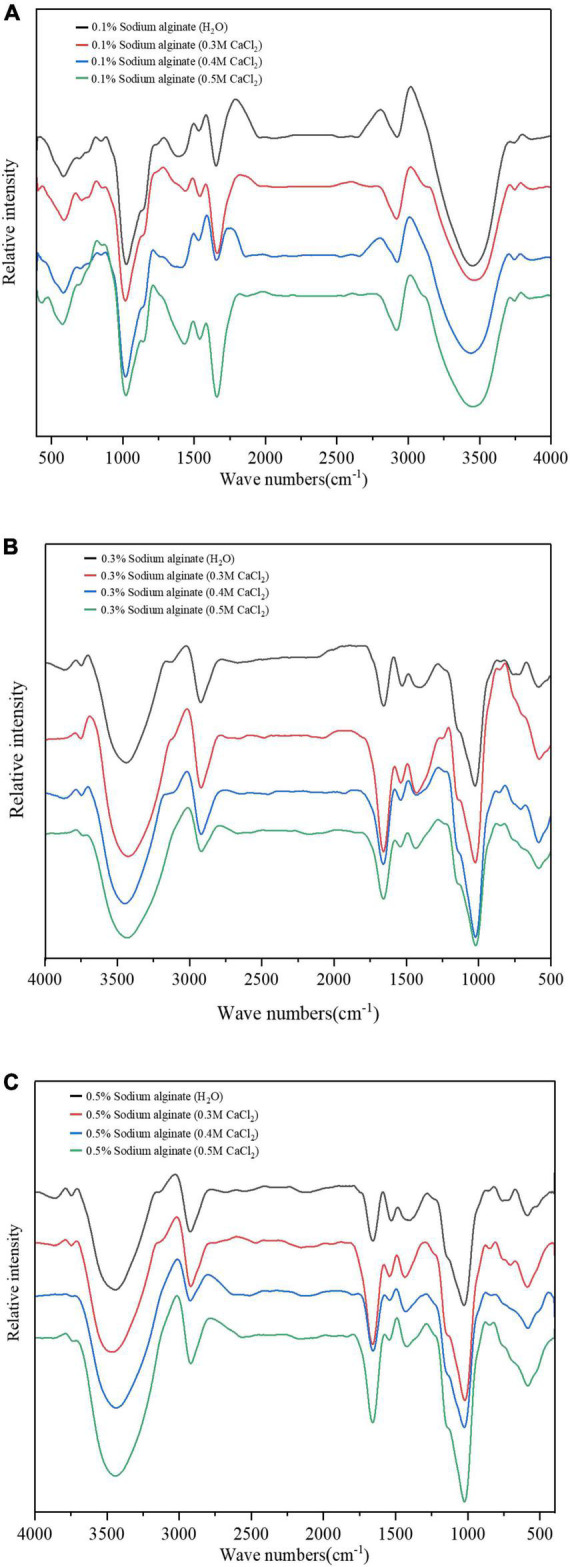
**(A–C)** Show the Fourier transform infrared spectra of sodium alginate with the addition of varying Ca^2+^ concentrations by the exogenous method.

The FTIR spectra in the wavenumber range of 399–4,000 cm^–1^ of starches with exogenously incorporated Ca^2+^ and sodium alginate are shown in Figure 6. As seen in the figure, the spectra of the different samples are similar. The absorption peak at 1,652 cm^–1^ results from the stretching vibration of C = O, also seen in α-helical structures of proteins (28). The absorption peak at 2,927 cm^–1^ results from the asymmetric stretching vibration of CH_2_ (29). Major absorption peaks are visible in the hydroxyl region (centered at 3,394-3,423 cm^–1^), likely the result of hydrogen bonds between the starch particles, alginate, and gel polysaccharide molecules (30). Compared with the sodium alginate only samples, the addition of calcium ions did not result in a new absorption peak, indicating that no chemical bonds were formed between sodium alginate and calcium in the noodles. The Fourier infrared spectrogram results of the endogenous method are similar to those of the exogenous method, with no significant changes, which are not mentioned here.

## 4. Conclusion

We prepared novel types of functional noodles by two simple methods and studied their properties in relation to cooking, texture, and digestion, as well as analyzing the structure of the starch. It was found that the stable cross-linking system formed by Ca^2+^ and sodium alginate could reduce the rate of water absorption by the noodles, and improve the textural properties of the noodles, such as hardness, springiness, cohesiveness, adhesiveness, and chewiness. In addition, the formation of the alginate network structure altered the area of the crystal zone of the noodles, although no new chemical bonds or substances were generated. Most importantly, the cross-linking of Ca^2+^ and sodium alginate significantly reduced the amount of glucose released from the noodles. In conclusion, the noodles prepared by the exogenous method were superior to those prepared using the endogenous method in terms of both noodle quality and lower glucose release, which will contribute to the development of functional foods. However, these results were obtained using simulated digestion *in vitro*, and further *in vivo* investigations are needed for verification.

## Data availability statement

The original contributions presented in this study are included in the article/supplementary material, further inquiries can be directed to the corresponding authors.

## Author contributions

BH, JY, and JC contributed to the conception of the study. HW performed the experiment, data analyses, and wrote the manuscript. JZ and LH contributed significantly to analysis and manuscript preparation. YZ helped perform the analysis with constructive discussions. All authors contributed to the article and approved the submitted version.
